# Sleep Duration among Children 8 Months after the 2011 Japan Earthquake and Tsunami

**DOI:** 10.1371/journal.pone.0065398

**Published:** 2013-05-30

**Authors:** Masahide Usami, Yoshitaka Iwadare, Masaki Kodaira, Kyota Watanabe, Momoko Aoki, Chiaki Katsumi, Kumi Matsuda, Kazunori Makino, Sonoko Iijima, Maiko Harada, Hiromi Tanaka, Yoshinori Sasaki, Tetsuya Tanaka, Hirokage Ushijima, Kazuhiko Saito

**Affiliations:** 1 National Center for Global Health and Medicine, Kohnodai Hospital, Department of Child and Adolescent Psychiatry, Ichikawa, Japan; 2 Saitama Prefecture Psychiatric Hospital, Department of Child and Adolescent Psychiatry, Saitama, Japan; 3 Kitasato University, Department of Psychiatry, Sagamihara, Japan; 4 Kumamoto University Graduate School of Medicine, Department of Psychiatry, Kumamoto, Japan; The University of Hong Kong, Hong Kong

## Abstract

**Background:**

To elucidate relationships between disaster damage conditions and sleep duration among children who survived the 2011 Japan earthquake and tsunami.

**Methods:**

The subjects comprised 12,524 children in kindergartens, elementary schools, and junior high schools in Ishinomaki City, Miyagi Prefecture, Japan. The Post Traumatic Stress Symptoms for Children 15 items (PTSSC-15), a self-completion questionnaire on traumatic symptoms, and a sleep questionnaire were distributed to them. A questionnaire regarding disaster damage conditions of the children’s homes was distributed to their teachers. Of 12,524, an effective response was obtained from 11,692 (93.3%).

**Results:**

Relationships between sleep duration and traumatic symptoms were displayed low correlations. Children with house damage and/or evacuation experiences slept for a significantly shorter time than children without these experiences.

**Conclusion:**

It is critical not only to examine traumatic symptoms in children but also to collect sleep duration and disaster damage conditions following natural disasters.

## Introduction

On March 11th, 2011, Japan experienced a huge earthquake and tsunami. The tsunami caused tremendous damage and victimized a number of children[1], [2].

To date, there have been many studies on children who have survived the disaster[1], [2], as well as on the psychiatric problems of children who experienced the 2004 Southeast Asian tsunami[3]–[11]. After any disaster, posttraumatic stress disorder (PTSD) is the psychiatric disease that should be considered most carefully[10]–[14]. Many studies have examined the relationships between PTSD and sleep problems such as nightmares, sleep disturbance, abnormalities in rapid-eye-movement sleep, arousal threshold during sleep, body movement during sleep, and breathing-related sleep disorders[15]–[20]. But there are few studies of the sleep problems in children who survived the 2011 tsunami[3], [21], [22], and these studies did not address sleep duration.

We collected information on the traumatic symptoms and sleep duration of children who experienced the earthquake and tsunami: gender, school grade, sleep duration, and environmental damage conditions. The aim of this study was to evaluate relationships between sleep duration and traumatic symptoms in children 10 months after the earthquake and tsunami, providing insight for future investigations of risk factors for traumatic symptoms after disasters[13], [23], [24].

## Methods

### Study design and setting

We performed an observational study of psychiatric symptoms among children after the 2011 Japanese earthquake and tsunami. Ishinomaki City is the second largest city (population, 162,822) in Miyagi Prefecture, Japan[2]. As of February 28, 2013, the death toll in Ishinomaki City was 3,147, and 448 were missing. The total number of collapsed houses and buildings, including half-collapsed houses, was 33,378; there were 7,298 temporary houses.

### Recruitment and participants

This survey was conducted as part of the school education program under the initiative of the Board of Education in Ishinomaki City. Surveys were distributed to all children who attended five kindergartens, 43 elementary schools, and 21 junior high schools in Ishinomaki City, Miyagi Prefecture. The survey was carried out in November 2011 (eight months after the earthquake disaster) after temporary houses had been provided for all evacuees in need in Ishinomaki City and after all evacuation centers had been closed.

First, the survey method was explained to the principals of all of the schools by the Education Committee of Ishinomaki City. Then teachers distributed a letter explaining the survey, which had been constructed by the Education Committee, to all children and their parents. The letter clearly stated that the students’ filling out the questionnaire would be considered for both the parents and the students as having given consent to the survey. The letter also specified that the survey results would be used to provide children with psychological care to facilitate their education at school and that the results would be published as a medical paper. Informed consent was obtained when the students filled out the questionnaire. This consent procedure was approved by the ethical committee of the National Center for Global Health and Medicine.

The Posttraumatic Stress Symptoms for Children 15 items (PTSSC-15), a self-completion questionnaire on traumatic symptoms, was distributed to 12,524 children registered at municipal schools in Ishinomaki City, and a questionnaire on the environmental damage experienced by the children was distributed to their teachers. Parents of kindergartners and first- to third-grade elementary school students were asked to fill out the questionnaire while talking with their children. Informed consent for participation in the survey was obtained at the time that the completed questionnaires were received from the children.

We received 12,346 (98.6%) of the 12,524 questionnaires distributed. Of the 12,346 children, effective response was obtained from 11,692 (93.3%). Answers for environmental damage with regard to all 12,524 children were returned from teachers.

### Measures

A paper-based survey was conducted, asking questions regarding traumatic symptoms and sleep duration using a self-report form.

The self-report form consisted of the PTSSC-15 and a sleep questionnaire developed by the authors. The teacher-report form consisted of a disaster situation questionnaire for each student, developed by the authors.

### PTSSC-15

The PTSSC-15 is a self-completion questionnaire on the stress reactions in children after disasters. The Posttraumatic Stress Symptom 10 (PTSS10) [25], which has fewer questions and was used as a screening test after the Hanshin Great Earthquake, is familiar in Japan[14]. In 105 Norwegian children (6–17 years old) devastated by the 2004 Southeast Asia Tsunami, PTSS10 was administered 10 and 30 months after the disaster[26]. Five questions that were considered to be important psychosomatic characteristics after disasters (that is, flashback, appetite loss, somatic reaction such as headache and abdominal pain, attention deficit, and anxiety) were added to the PTSS10, and the PTSSC-15, consisting of 15 questions, was constructed and validated in Japan[27].

Each question is scored in six levels: 0 =  completely disagree, 1 =  mostly disagree, 2 =  partially disagree, 3 =  partially agree, 4 =  mostly agree, and 5 =  completely agree. Higher scores indicate more severe trauma and depressive symptoms.

### Sleep questionnaire

The Sleep questionnaire included items related to waking and sleep-onset times. For total sleep duration on weekdays and holidays, the subject was asked to write the total usual daily sleep hours, usual wake-up time, and the time that the subject usually goes to sleep.

### Disaster damage

The questionnaire regarding disaster damage experienced by the subject children employed a format to be filled in by teachers, and the contents addressed the condition of disaster damage, presence or absence of bereavement experience, and life in evacuation centers.

With regard to the condition of disaster damage of the child’s house, one of the following three answers was selected: “no damage,” “total collapse by the earthquake or tsunami (incapable of living in the house),” or “half collapse by the earthquake or tsunami (necessary to repair the house to live in it).” A huge tsunami struck in Ishinomaki City. A new seaside residential area had experienced especially tremendous damage. Many children of young married couples lived in the area.

Regarding the living conditions in evacuation centers, multiple-answer questions and answers were selected from the following status: “no experience,” “currently living in the evacuation center,” “used to live in the evacuation center,” “living in a temporary house,” and “used to live in a temporary house.” Victims were able to reside from a few weeks to several months at evacuation centers. However, in Ishinomaki City, elementary and junior high schools in the region also became emergency evacuation centers. Some evacuation centers were packed with more than 1,000 victims. Many evacuation centers could not provide electricity or water. Therefore, sanitary conditions in shelters were poor. It was also difficult to protect the privacy of victims, making them uncomfortable.

As to the bereavement experience (including the experience of unexplained disappearances caused by the earthquake), multiple answers were allowed from the following eight responses: “no experience,” “father,” “mother,” “brothers and sisters,” “grandfather and grandmother,” “kindergarten and school classmates at the time of the earthquake,” “teacher in charge of the class at the time of the earthquake,” and “others.”

### Statistical analysis

The average waking and sleep-onset times on weekdays and holidays in children were calculated for each grade and gender. The average sleep duration on weekdays and holidays in children was also calculated for each grade and gender. Correlations were calculated between total PTSSC-15 score, age, grade, and sleep duration of children suffering from traumatic experiences.

Furthermore, with regard to the relationship between disaster experience and sleep duration, children were divided into two groups with and without disaster experiences (house damage, evacuation experiences, or bereavement experiences). The average sleep duration on weekdays and holidays in male children and female children were statistically compared with the disaster damage conditions by two-factor analysis of variance.

In all tests, a significance level of 0.05 was used in two-sided tests. Analyses were performed using PASW 18.0.

## Results

### Descriptive information

The participants included 11,692 children (5959 males, 5733 females) who were exposed to the huge 2011 Japanese earthquake and tsunami.

Table 1 shows the gender, age, PTSSC-15 score, and disaster damage conditions (house damage, evacuation conditions, and bereavement experience) in 11,692 children. When teachers had no information about house damage, evacuation conditions, and bereavement experience, the answer was defined as “unknown.”

**Table 1:** Characteristics of children who experienced the 2011 Japan Earthquake and Tsunami.

Items		n = 11639	
Gender	Male	5939	(51.0%)
	Female	5700	(49.0%)
Age at the time of the disaster (y) (Mean)		10.9	(SD = 2.7)
PTSSC-15 score (Mean)		20.5	(SD = 14.5)
Sleep duration	Weekdays (Mean)	8.5	(SD = 1.2)
	Holidays (Mean)	9.0	(SD = 1.4
Eating breakfast	Yes	827	(7.1%)
	No	10673	(91.7%)
	Unknown	139	(1.2%)
House damage	None	6986	(60.0%)
	Total collapse	2243	(19.3%)
	Half collapse	2354	(20.2%)
	Total	4597	(39.5%)
	Unknown	56	(0.5%)
Evacuation experience	None	8228	(70.7%)
	Currently living in evacuation center	90	(0.8%)
	Used to live in evacuation center	2845	(24.4%)
	Living in temporary housing	976	(8.4%)
	Used to live in temporary housing	51	(0.4%)
	Evacuation experience at least once	3248	(27.9%)
	Unknown	163	(1.4%)
Bereavement experience	None	9241	(79.4%)
	Father	71	(0.6%)
	Mother	66	(0.6%)
	Brothers and sisters	44	(0.4%)
	Grandfather and grandmother	355	(3.1%)
	Classmates	1498	(12.9%)
	Teacher in charge	32	(0.3%)
	Others	270	(2.3%)
	At least one bereavement experience	2103	(18.1%)
	Unknown	295	(2.5%)

Table 2 and Table 3 show waking and sleep-onset times for each grade and gender on weekdays and holidays. Table 4 shows sleep durations for each grade and gender on weekdays and holidays.

**Table 2:** Waking and sleep-onset times on weekdays.

Grade	Waking time						Sleep-onset time					
	Male			Female			Male			Female		
	M	SD	N	M	SD	N	M	SD	N	M	SD	N
1^st^	6:48	0.6	579	6;48	0.5	546	21:24	0.7	579	21.4	0.7	546
2^nd^	6:48	0.4	602	6;54	0.5	525	21:30	0.7	602	21.6	0.7	525
3^rd^	6:30	0.4	640	6;30	0.5	618	21:42	0.7	640	21.8	0.7	618
4^th^	6:30	0.4	649	6;24	0.5	674	22:06	1.1	649	22.0	0.9	674
5^th^	6:30	0.5	625	6;30	0.4	612	22:12	1.1	625	22.3	0.9	612
6^th^	6:18	0.6	699	6;24	0.5	683	22:30	1.0	699	22.6	1.0	683
7^th^	6:24	0.6	646	6;24	0.6	586	23:00	1.2	646	23.0	1.2	585
8^th^	6:24	0.6	667	6;24	0.5	609	23:24	1.4	667	23.5	1.2	608
9^th^	6:24	0.6	653	6;30	0.6	658	24:06	1.5	651	24.1	1.3	660

M: mean, SD: Standard Deviation (Hour), N: Number,

**Table 3:** Waking and sleep-onset times on holidays.

Grade	Waking time						Sleep-onset time					
	Male			Female			Male			Female		
	M	S	N	M	SD	N	M	SD	N	M	SD	N
1^st^	6:54	0.8	579	7.2	0.8	546	21:24	0.7	579	21.4	0.7	546
2^nd^	6:48	0.9	602	7.3	0.8	525	21:30	0.7	602	21.6	0.7	525
3^rd^	6;54	1.1	640	7.4	1.1	618	21:42	0.7	640	21.8	0.7	618
4^th^	6:36	1.6	649	7.5	1.3	674	22:06	1.1	649	22.0	0.9	674
5^th^	6:48	1.6	625	7.7	1.3	612	22:12	1.1	625	22.3	0.9	612
6^th^	7:06	1.4	699	7.9	1.3	683	22:30	1.0	699	22.6	1.0	683
7^th^	7:30	1.6	646	8.1	1.4	586	23:00	1.2	646	23.0	1.2	585
8^th^	7:56	1.8	667	8.3	1.5	609	23:24	1.4	667	23.5	1.2	608
9^th^	8:30	1.7	653	8.8	1.5	658	24:06	1.5	651	24.1	1.3	660

M: mean, SD: Standard Deviation (Hour), N: Number,

7^th^ –9^th^:1^st^–3^rd^ grade of junior high school students.

**Table 4:** Sleep durations on weekdays and holidays.

Grade	Weekdays						Holidays					
	Male			Female			Male			Female		
	M	S	N	M	SD	N	M	SD	N	M	SD	N
1^st^	9.4	0.6	577	9.4	0.6	544	9.5	0.8	577	9.8	0.7	545
2^nd^	9.3	0.6	601	9.3	0.6	525	9.3	1.0	602	9.7	0.8	523
3^rd^	9.2	0.6	639	9.2	0.6	617	9.2	0.9	637	9.6	0.9	610
4^th^	9.0	0.9	647	9.0	0.7	672	8.5	1.5	644	9.4	1.2	653
5^th^	8.7	0.9	626	8.7	0.8	610	8.4	1.5	607	9.4	1.2	606
6^th^	8.5	0.9	697	8.4	0.8	682	8.6	1.4	693	9.3	1.3	673
7^th^	7.9	1.0	641	7.7	1.0	586	8.5	1.5	637	9.0	1.3	575
8^th^	7.7	1.2	663	7.4	1.0	609	8.4	1.6	651	8.7	1.4	599
9^th^	7.2	1.2	651	6.9	1.1	657	8.4	1.6	644	8.6	1.5	651

M: mean(Hour), SD: Standard Deviation (Hour), N: Number,

7^th^ –9^th^:1^st^–3^rd^ grade of junior high school students.

### Traumatic symptoms and sleep duration

Correlations between PTSSC-15, age, grade, and sleep durations on weekends and holidays are shown in Table 5 and Table 6. The results show a high correlation between Grade and Sleep duration. Furthermore, the average sleep duration on weekdays and holidays is shown in Table 7 and Table 8, respectively, for each gender. Sleep duration on weekdays was significantly shorter in children with house damage or evacuation experience than in children without these experiences (F(1,11424)  = 12.47, P = 0.0004; F(1,11319)  = 75.92, P<0.0001). In addition, sleep duration on holidays was significantly shorter in children with evacuation experiences than in children without these experiences (F(1,11205)  = 5.459, P<0.0001).

**Table 5:** Correlations between PTSSC-15, grade, and sleep durations on weekends and holidays in male children.

	PTSSC-15	Grade	Sleep duration	Sleep duration
			(Weekday)	(Holiday)
PTSSC-15		0.106	–0.093	–0.032
Grade			–0.661	–0.274
Slee duration		–		0.395
(Weekday)				
Sleep duration				
(Holiday)				

All correlations were significant at p<0.001.

**Table 6:** Correlations between PTSSC-15, grade, and sleep durations on weekends and holidays in female children.

	PTSSC-15	Grade	Sleep duration	Sleep duration
			(Weekday)	(Holiday)
PTSSC-15		0.212	–0.182	–0.096
Grade			–0.723	–0.321
Slee duration				0.428
(Weekday)				
Sleep duration				
(Holiday)				

All correlations were significant at p<0.001.

**Table 7:** Average sleep duration on weekdays (number of disaster experiences and gender).

Disaster experiences	Sleep duration on weekdays							F	p value
	Male			Female					
	M	SD	N	M	SD	N			
Children with house damage							Gender × house damage	1.754	ns
No	8.56	1.16	3496	8.49	1.17	3384	Gender	19.49	<0.0001
Yes	8.51	1.18	2336	8.38	1.25	2212	House damage Gender	12.47	0.0004
Children who experienced evacuation at least once							Gender × evacuation experience	0.37	ns
No	8.59	1.14	4161	8.51	1.17	3944	Gender	14.82	0.0001
Yes	8.39	1.23	1613	8.28	1.28	1605	Evacuation experience	75.92	<0.0001
Children experiencing bereavement							Gender × bereavement experience	1.456	ns
No	8.54	1.17	4687	8.46	1.19	4428	Gender	15.72	<0.0001
Yes	8.54	1.21	1028	8.39	1.28	1047	Bereavement experience	1.456	ns

**Table 8:** Average sleep duration on holidays (number of disaster experiences and gender).

Disaster experiences	Sleep duration on holidays							F	p value
	Male			Female					
	M	SD	N	M	SD	N			
Children with house damage							Gender × house damage	0.6131	ns
No	8.77	1.41	3469	9.27	1.23	3347	Gender	414.5	<0.0001
Yes	8.73	1.42	2313	9.27	1.24	2183	House damage Gender	0.6131	ns
Children who experienced evacuation at least once							Gender × evacuation experience	0.8076	ns
No	8.78	1.41	4127	9.28	1.21	3897	Gender	356.1	0.0001
Yes	8.69	1.42	1599	9.24	1.29	1586	Evacuation experience	5.459	<0.0001
Children experiencing bereavement							Gender × bereavement experience	0.02	ns
No	8.75	1.43	4650	9.27	1.23	4377	Gender	259.00	<0.0001
Yes	8.74	1.36	1016	9.27	1.27	1033	Bereavement experience	0.02	ns

## Discussion

This study elucidated sleep durations in children who suffered from tremendous disaster-related trauma. Relationships between sleep duration and traumatic symptoms were displayed low correlations. However, children with house damage and/or evacuation experiences slept for a significantly shorter time than children without these experiences.Sleep duration was related with gender, school grade, house damage, evacuation experience.

If psychiatrists or clinical psychologists use a self-completion questionnaire as a screening tool for PTSD after a disaster, they are obligated to treat a number of children labelled at high risk for PTSD[2], [27]. Therefore, a self-completion questionnaire is insufficient as a screening tool for PTSD after a disaster. Other clinical information related to PTSD must be collected to target those at highest risk. The self-completion questionnaire would label more children at high risk for PTSD than could possibly be treated; therefore, other screening tools must be used to reduce the population that requires treatment to provide help to those who most need it.

Because sleep duration was related to disaster damage conditions, it is reasonable to suggest the importance of sleep duration for the mental health of children after a disaster.

Our study has some limitations. The relationships between sleep duration and traumatic symptoms were insignificant and exhibited low correlations. Also, the recorded differences between individual sleep durations was rather small, as all were within an hour. Because the qualitative sleeping problems and sleeping habits before the earthquake were not determined, qualitative problems in sleeping habits and the influence of the earthquake cannot be clarified. This survey was performed in only one district in Japan, and it is impossible to evaluate qualitative and quantitative aspects of sleep in children after the 2011 Japanese earthquake and tsunami on the basis of the survey results. Therefore, this study is insufficient as an epidemiological survey for psychiatric diagnosis. Examinations by child psychiatrists using operational diagnostic criteria, structured interviews, activity monitoring devices, and night-time polysomnography are necessary for accurate psychiatric diagnosis. Moreover, the results of this study on children in Ishinomaki City do not reflect all characteristics of children experiencing the 2011 Japanese earthquake and tsunami.

In studies on children after disaster damage, it is important to evaluate trauma symptoms and sleep duration in addition to the direct experiences of the disaster such as evacuation conditions and bereavement experience. Importantly, the large sample size in the present study allowed us to draw clinically valuable conclusions.

## Conclusions

This study elucidated relationships between disaster-related damage conditions and sleep duration in children who survived the 2011 Japanese earthquake and tsunami. It is important not only to evaluate the traumatic symptoms with a self-completion questionnaire but also to confirm sleep duration and damage conditions after the disaster.
